# Significance, Fragility, and Robustness in Clinical Trials: Stratifying Statistical Evidence

**DOI:** 10.7759/cureus.100494

**Published:** 2025-12-31

**Authors:** Thomas F Heston

**Affiliations:** 1 Family Medicine, University of Washington, Seattle, USA; 2 Medical Education and Clinical Sciences, Washington State University, Spokane, USA

**Keywords:** clinical trial methodology, fragility index, modified arm fragility quotient, monte carlo simulation, neutrality boundary framework, risk quotient, statistical robustness, trial evidence quality

## Abstract

Background

Current reporting standards treat p-values, effect sizes, and confidence intervals as complete evidence, but this is only partial: it quantifies significance and magnitude, not classification stability (fragility) or distance from therapeutic neutrality (robustness). This study validates the p-fr-nb framework in two-arm, binary-outcome clinical trials. Framework extensions to continuous, ordinal, survival, and correlation analyses exist but are not empirically validated here. For this study, the p-fr-nb triplet is defined as providing the p-value (significance), fragility (classification stability), and robustness (distance from neutrality) in trial results. This triplet assesses completeness across three statistical inferential dimensions; it stratifies evidence quality but does not prove truth, causality, or replication.

Methodology

A pragmatic observational validation study of two-arm, binary-outcome clinical trials identified in PubMed (n = 129 across 15 specialties) was conducted. Null expectations were generated with a Monte Carlo simulation of 720,000 trials across 360 design scenarios, including 120,000 null trials (true relative risk (RR) = 1.0). Simulations represent unfiltered random trial generation and do not model publication bias or selective reporting. Fragility was measured by the modified-arm fragility quotient (MFQ; fragility index divided by the size of the modified arm). Robustness was measured by the risk quotient (RQ), defined for 2×2 tables as RQ = |ad − bc| / (N²/4). Concordant-positive (CP) evidence was defined as p ≤ 0.05, MFQ > 0.10, and RQ ≥ 0.227, with the RQ cutoffs based on large-scale simulation. The significant-fragile-weak (SFW) pattern was defined as p ≤ 0.05, MFQ ≤ 0.10, and RQ < 0.075. The main outcomes were the rates of CP and SFW among statistically significant empirical trials, compared with null-simulation expectations.

Results

In null simulations (RR = 1.0), the CP triplet occurred in 1.4% of significant trials; even with strong effects (RR = 0.60), it appeared in only 4.7%. Of the 129 trials analyzed, 77 (59.7%) were statistically significant. Among these 77 trials, 30 (39.0%; 95% confidence interval = 28.8-50.1%) met the CP criteria, a 27.9-fold higher compared with null expectations (p < 0.0001). Overall, 61.0% of significant trials were fragile (MFQ ≤ 0.10, 47/77), 31.2% were weakly robust (RQ < 0.075, 24/77), and 31.2% showed the SFW pattern (24/77).

Conclusions

In this heterogeneous sample, the p-fr-nb framework stratified positive findings beyond what p-values and confidence intervals reveal. Among 77 significant trials, 39.0% met stringent criteria for stability and strong robustness, a distinction not visible from p-values alone. Conversely, 31.2% showed the SFW pattern, where significance was fragile, and separation from no effect was minimal. Fragility and robustness metrics provide interpretive dimensions not captured by p-values alone, enhancing assessment of evidence heterogeneity relevant to reproducibility and clinical interpretation. These data support further evaluation of incorporating fragility and robustness metrics into the reporting of clinical trial results.

## Introduction

The replication crisis in biomedical research has exposed fundamental limitations in how statistical evidence is evaluated [1,2]. Central to this crisis is the near-exclusive reliance on p-values to determine whether trial results are “statistically significant” (p ≤ 0.05) or “not statistically significant” (p > 0.05). While p-values quantify the compatibility of observed data with the null hypothesis, they provide only partial statistical evidence because they address only one statistical dimension: the probability of observing data at least as extreme as those obtained if no treatment effect exists [3].

Two critical questions remain unanswered by p-values alone. First, how stable is the significance classification? Consider two trials of identical size reporting a p-value of 0.04, but flipping two outcomes reverses significance in one trial, while 10 outcome changes are required in the other. P-values alone do not show this difference in fragility; these two statistical dimensions are separate yet complementary.

Second, how far are the observed data from therapeutic neutrality, where the intervention has no effect? Confidence intervals do convey distance from the null hypothesis and provide information about precision and plausible effect size ranges. However, they do not offer a standardized 0-1 scale that enables systematic cross-study comparison or objective classification of robustness across diverse study designs. Robustness metrics complement confidence intervals by providing this normalized scale.

Current reporting standards emphasize p-values, effect sizes, and confidence intervals [4,5]. While effect sizes quantify magnitude in original units and confidence intervals quantify precision, neither directly measures classification stability (fragility) or provides a standardized 0-1 scale for geometric separation from neutrality (robustness) that applies uniformly across diverse study designs. These limitations have been recognized for decades, yet p-value-centered reporting persists as standard practice [6,7].

Two separate dimensions: fragility and robustness

A critical conceptual clarification is required before introducing this framework. In this study, robustness is not the opposite of fragility. In common usage, “robust” and “fragile” are often antonyms, suggesting that measuring one eliminates the need for the other. In this framework, they measure orthogonal dimensions of evidence quality.

*Fragility* quantifies classification stability: the minimum change in outcomes required to flip the significance decision across the p = 0.05 threshold. It answers: “How resistant is this p-value to small outcome changes?”

*Robustness* quantifies how far the observed result lies from the neutrality boundary on a 0-1 scale. This differs from effect size, which quantifies magnitude in original units, and from confidence intervals, which quantify precision and plausible range. Robustness combines effect size with variability to answer, in essence, “how real is the evidence?”

Weak robustness signals that the data remain compatible with negligible real-world benefit despite a significant p-value, seemingly favorable effect size, or narrow confidence intervals. Strong robustness supports the hypothesis that the treatment has a true effect, although the effect size is still required to judge clinical utility.

Fragility and robustness are closely related but answer fundamentally different questions. A result can be fragile yet show strong robustness, suggesting an underpowered study detecting a real effect. Findings that are stable but weakly robust are typical in large, well-powered studies that detect trivial effects. Results that are both fragile and weakly robust raise concerns about reproducibility, as small changes in outcomes could eliminate statistical significance while the effect remains near neutrality. Conversely, results that are both stable and strongly robust represent high-quality evidence. These patterns reveal heterogeneity in evidence quality that significance testing alone cannot distinguish.

The four evidence dimensions are distinguished as follows: *Fragility* measures classification stability (how many outcome changes flip significance). *Robustness* measures distance from neutrality on a standardized 0-1 scale (how far from no-effect). *Effect size* measures the magnitude in original units (how large the difference is). *Precision* measures uncertainty via confidence intervals (the plausible range).

Fragility and robustness are statistical inferential dimensions; effect size and precision quantify magnitude and uncertainty. All four contribute to evidence interpretation but measure fundamentally different properties.

Prior approaches to assessing evidence stability

The fragility index (FI) quantifies classification stability by measuring the minimum number of outcome changes in a specified trial arm required to reverse statistical significance [8]. Subsequent refinements include the fragility quotient (FQ), which normalizes to total sample size, and the modified-arm fragility quotient (MFQ), which adjusts for imbalanced allocation [9,10]. However, fragility metrics capture only one dimension, how easily the significance label flips, and cannot distinguish between underpowered studies detecting real effects versus well-powered studies detecting trivial effects. Fragility quantifies the stability of the decision boundary crossing but does not measure how far the point estimate lies from therapeutic neutrality.

The research gap: partial statistical evidence

Existing fragility studies report FI or FQ distributions but do not incorporate explicit measures of distance from neutrality [11]. Effect size reporting provides magnitude estimates, and confidence intervals incorporate variability to quantify precision. However, existing standards do not provide a single normalized metric that combines effect size with variability to measure distance from neutrality on a standardized 0-1 scale comparable across diverse study designs. Robustness addresses this gap by quantifying distance from neutrality as a normalized signal-to-noise ratio, the observed effect relative to its variability on a standardized 0-1 scale.

The neutrality boundary framework (NBF) addresses this gap by quantifying robustness, geometric distance from neutrality, on a 0-1 scale, and assigning it to the summary statistic “nb” [12]. For independent-sample binary or multinomial outcome trials, the risk quotient (RQ) provides the value of nb. The underlying formula for nb is broadly generalizable. It equals the absolute distance between the observed statistic and its neutrality value, divided by that distance plus a fixed reference distance that sets the scale for the statistic, thereby normalizing nb to lie between 0 and 1. This same construction can be extended to the full range of clinical trial designs, including analysis of variance (ANOVA), continuous outcomes, and survival analyses. The NBF, via nb, enables standardized robustness assessment regardless of study structure.

The p-fr-nb framework

Current guidelines emphasize p-values, effect sizes, and confidence intervals. While these are important, they do not directly measure classification stability or provide a standardized metric for distance from neutrality across diverse study designs. The p-fr-nb framework addresses this gap by providing three complementary dimensions: the p-value (significance), fr (classification stability), and nb (distance from neutrality). This framework provides a standardized, model-free way to assess significance, fragility, and robustness using 0-1 scales to enable cross-study comparisons. The p-fr-nb triplet, combined with effect size and 95% CIs, provides a more comprehensive statistical analysis for research findings.

Applying this framework to independent-sample binary outcome trials in the present analysis: p-values capture significance (compatibility with the null) on a 0-1 scale; the MFQ captures fragility (classification stability) on a 0-1 scale; and the RQ captures robustness (distance from neutrality) on a 0-1 scale.

Study objectives

This study had three aims: first, to establish null-hypothesis expectations for the prevalence of concordant-positive (CP) evidence, simultaneous significance, stability, and strong robustness, using large-scale Monte Carlo simulation; second, to estimate the prevalence of CP evidence within the p-fr-nb framework in published two-arm, binary-outcome clinical trials across diverse specialties; and third, to estimate the prevalence of discordant evidence, defined as the significant-fragile-weak (SFW) pattern, in the same trials. These objectives were designed to evaluate the added value of the p-fr-nb triplet as a complement to conventional p-value-centered analysis of clinical trials.

This study hypothesizes that (a) the prevalence of CP evidence in published significant trials would substantially exceed null expectations, and (b) fragility-robustness patterns, including the SFW pattern, would reveal important heterogeneity in evidence quality that is not revealed by p-values alone.

## Materials and methods

Study design and reporting standards

A pragmatic observational study was conducted to empirically validate the p-fr-nb framework by demonstrating its ability to discriminate between evidence quality patterns in published binary-outcome clinical trials. This validation is empirical (applying the framework to real trial data) rather than criterion validation (comparison to a gold standard) or predictive validation (forecasting replication outcomes). The study design followed Statistical Analyses and Methods in the Published Literature (SAMPL) guidelines for reporting statistical methods and results [13].

Trial selection and data collection

This study evaluates the p-fr-nb framework’s ability to discriminate evidence quality patterns, not to estimate population prevalence of fragility or robustness. Clinical trials were identified through pragmatic convenience sampling prioritizing diversity: trials were drawn from prior fragility analyses, PubMed searches (often ‘clinicaltrials.gov[Title/Abstract]’ sorted by trending/recent), and targeted clinical domain searches. Each trial underwent manual screening to confirm a binary outcome that could be analyzed as an independent-sample 2×2 table with sufficient data. Primary and secondary outcomes were treated equivalently. This diversity-focused approach enables demonstration that the framework discriminates patterns across heterogeneous contexts; it does not support inferences about population prevalence in specific clinical domains.

Inclusion criteria were: (1) clinical trial design; (2) binary primary or secondary outcome that could be represented as a 2×2 contingency table; (3) sufficient data to calculate fragility and robustness metrics (cell counts or equivalent summary statistics); (4) independent-sample design (matched-pair and crossover designs excluded). No restrictions were placed on publication date, journal impact factor, trial phase, or disease area.

Data were extracted from each trial, including 2×2 contingency table cell counts (a, b, c, d) and clinical domain. P-values were recalculated uniformly from extracted cell counts rather than extracted from publications. The FI, MFQ, and RQ were calculated from the cell counts using the binary_2x2_independent.ipynb calculator, available on the Fragility Metrics Toolkit [14]. P-values were calculated using the two-sided Fisher’s exact test for most trials. For trials with a total sample size exceeding 5,000 participants and with all 2×2 table cells containing at least 50 observations, Pearson’s chi-square test was used for computational efficiency. Recalculated p-values may differ slightly from originally published values due to different test selections or continuity corrections.

Monte Carlo simulation design

To establish null and non-null expectations, a large-scale Monte Carlo simulation generating 720,000 binary outcome trials under known data-generating parameters was performed. The simulation crossed five design factors: (a) sample sizes (N): 60, 100, 200, 400, 800; (b) allocation ratios: 1:1, 2:1, 3:2; (c) control event rates: 0.05, 0.10, 0.20, 0.40; (d) true relative risks: 1.00 (null), 0.90, 0.80, 0.70, 0.60, 1.10; and (e) replications per scenario: 2,000. This design yielded 360 unique scenarios (5 sample sizes × 3 allocations × 4 control rates × 6 effect sizes), with 2,000 replications each. The null-hypothesis subset (RR = 1.00) comprised 120,000 trials, while the non-null scenarios (RR ≠ 1.00) comprised 600,000 trials.

Each simulated trial calculated p-values (Fisher’s exact test for N ≤ 50 or any cell <5; Pearson chi-square otherwise), MFQ, and RQ using the methods below. These simulations represent unfiltered random trial generation and do not model publication bias, trial stopping rules, or selective reporting; they establish benchmark expectations for what random data generation produces under known effect sizes. The Monte Carlo simulation code and complete results are archived on Zenodo [15].

Statistical metrics

Modified-Arm Fragility Quotient

Fragility was quantified using the MFQ, defined as the proportion of patients in a single study arm whose outcomes must change to reverse statistical significance [10]. MFQ is calculated as MFQ = FI / n_mod, where FI is the classic fragility index [8], and n_mod is the size of the arm subjected to toggling (typically the fewer-events arm; smaller arm in case of tie). Per the FI procedure, outcomes were first toggled one at a time until the p-value crossed the 0.05 threshold (in either direction), then divided by the number of subjects in that trial arm. MFQ ranges from 0 (maximally fragile) to 1 (maximally stable), with higher values indicating greater classification stability. Fragile results were defined as MFQ ≤ 0.10 and stable results as MFQ > 0.10. This threshold is heuristic and was selected to maintain conceptual symmetry with the conventional 5% significance level: for 1:1 allocation trials, MFQ = 0.10 (10% of one arm must change) equals FQ = 0.05 (5% of the total sample must change). Empirical exploration of the MFQ distribution confirmed that 0.10 provided effective discrimination between fragile and stable classifications without classifying nearly all trials as fragile. This threshold should not be interpreted as a universal normative standard; appropriate thresholds may vary by clinical context and study objectives.

Risk Quotient

Robustness was quantified using RQ. For 2×2 tables with cells {a, b, c, d}, and total sample size N, RQ is calculated as: RQ = |ad − bc| / (N²/4). RQ lies on a 0-1 scale representing geometric distance from neutrality, with higher values indicating stronger evidence of separation from the neutrality boundary.

Thresholds for RQ were established from an independent prior Monte Carlo simulation of 1 million trials using realistic clinical trial parameters: sample sizes 30-1,200, event rates 0.03-0.60, and relative risks 0.20-3.0 [14]. The 33rd and 67th percentiles of that simulated RQ distribution (≈0.075 and ≈0.227) were used to demarcate weak, moderate, and strong robustness categories. These tertile-based thresholds are heuristic; alternative percentile choices would shift category boundaries but not alter the primary finding of enrichment for high robustness in empirical trials compared with null expectations.

Concordant Statistical Evidence Pattern

For positive findings, a CP pattern within the p-fr-nb framework was defined as the simultaneous occurrence of statistical significance (two-sided p ≤ 0.05), stability (MFQ > 0.10), and strong robustness (RQ ≥ 0.227). This triplet identifies significant trials that require more than a 10% change in outcomes in one arm to reverse significance and lie in the highest robustness tertile of the neutrality boundary scale, indicating a far separation from neutrality.

For nonsignificant findings, a concordant-null pattern within the p-fr-nb framework was defined as the simultaneous occurrence of statistical nonsignificance (two-sided p > 0.05), stability (MFQ > 0.10), and weak robustness (RQ < 0.075). This triplet identifies nonsignificant trials that require more than 10% of one arm to change outcomes to reverse significance, and that lie in the lowest robustness tertile of the neutrality boundary scale (i.e., findings are close to therapeutic neutrality).

Significant-Fragile-Weak Pattern

For interpretation, the SFW boundary pattern was tracked among significant trials, defined as p ≤ 0.05, MFQ ≤ 0.10, and RQ < 0.075. SFW patterns represent discordant statistical findings: results that achieve statistical significance yet remain fragile (small outcome changes reverse significance) and weakly robust (observed data lie close to neutrality). This combination identifies trials where significance may not reflect a meaningful, replicable effect.

Statistical analysis

For the Monte Carlo simulation, the concordant-positive prevalence among significant trials (p ≤ 0.05) for each true RR value, providing benchmark expectations under both null and non-null scenarios, was calculated. The prevalence of the SFW pattern among significant trials was summarized.

For empirical trials, the CP pattern prevalence among statistically significant trials and concordant-null pattern prevalence among nonsignificant trials were calculated, both with 95% confidence intervals (CIs) using the Wilson score method for binomial proportions. Comparison was made of the observed CP prevalence to the null expectation established by simulation using exact binomial tests, and calculated fold elevation as the ratio of the empirical prevalence to the null expectation. Component prevalences were calculated as the proportion of significant trials exhibiting fragility (MFQ ≤ 0.10), weak robustness (RQ < 0.075), and the SFW pattern. Descriptive statistics (median and interquartile range) for MFQ and RQ were calculated stratified by significance status.

All analyses were conducted using R version 4.3.1 [16]. Two-sided p-values ≤0.05 were considered statistically significant for hypothesis tests.

Ethics statement

This study does not constitute human subjects research as defined by 45 CFR 46.102(e) because it involves re-analysis of aggregate statistical data from previously published, publicly available studies and does not involve obtaining information from living individuals or accessing identifiable private information. No institutional review board approval was required.

## Results

Trial characteristics

In total, 129 clinical trials met the inclusion criteria. Trials spanned 15 clinical specialties, with the largest representation from endocrinology (26.4%), autoimmune diseases (15.5%), and cardiology (14.0%). Other specialties included oncology (8.5%), infectious diseases and vaccines (8.5%), neurology, behavioral health, hepatology, nephrology, laboratory medicine, obstetrics and gynecology, pediatrics, pulmonology, and surgery (combined 27.1%). Sample sizes ranged from 12 to 332,438 participants (median = 194, IQR = 70-1,433). Trial allocation ranged from 1:1 to 23:1. In total, 21 (16.3%) trials used exact 1:1 allocation, with a median allocation ratio of 1.05:1 (IQR = 1.0-1.4). Of the 129 trials, 77 (59.7%) were statistically significant (p ≤ 0.05), and 52 (40.3%) were nonsignificant (p > 0.05). Trial characteristics are summarized in Table 1.

**Table 1:** Characteristics of 129 clinical trials. IQR = interquartile range

Characteristic	Value
Specialties represented	15
Sample size, range	12 to 332,438
Sample size, median (IQR)	194 (70-1,433)
Allocation ratio, range	1:1 to 23:1
Allocation ratio, median (IQR)	1.05 (1.0-1.4)
Statistically significant	77 (59.7%)

Monte Carlo simulation results

The Monte Carlo simulation generated 720,000 binary outcome trials across 360 design scenarios. Among the 120,000 null trials (true RR = 1.00), 4,783 (4.0%) achieved statistical significance (p ≤ 0.05). Of these, 65 met the CP criteria, yielding a prevalence of 1.4% (95% CI = 1.1-1.7%) among significant null trials.

Among simulations with non-null effects, CP prevalence among significant trials increased with effect size but remained modest: RR = 0.90 (1.5%), RR = 0.80 (1.6%), RR = 0.70 (2.6%), RR = 0.60 (4.7%), and RR = 1.10 (1.3%). Even the strongest simulated effect (RR = 0.60, a 40% risk reduction) yielded the CP pattern in only 4.7% of significant trials.

The relatively low prevalence, even under strong effects, reflects the stringent nature of the CP criteria: trials must simultaneously achieve statistical significance, maintain stability, and demonstrate strong geometric separation.

SFW prevalence among significant trials showed a different pattern: SFW occurred in 24.0% (RR = 0.60), 25.6% (RR = 0.70), 28.8% (RR = 0.80), 31.8% (RR = 0.90), 31.7% (RR = 1.0 null), and 28.4% (RR = 1.10) of significant trials. This flat distribution across effect sizes confirms that SFW is a boundary phenomenon, not a discriminator between true and null effects. Once a trial achieves p ≤ 0.05, approximately 24-32% will naturally lie near both the significance and neutrality boundaries regardless of the underlying effect magnitude. Simulation validation results are summarized in Table 2.

**Table 2:** Monte Carlo simulation results. RR = relative risk; CP = concordant-positive pattern; SFW = significant-fragile-weak pattern

Parameter	Value
Total simulated trials	720,000
Design scenarios	360
CP, null trials (RR = 1.0)	1.4%
CP, moderate effect (RR = 0.8)	1.6%
CP, strong effect (RR = 0.6)	4.7%
SFW pattern, range across all effect sizes	24-32%

The substantial difference between simulation expectations (1.4% CP prevalence under null) and empirical observations (39.0% CP prevalence) reflects the collective outcome of trial design, conduct, analysis, and publication processes. The p-fr-nb framework measures this enrichment pattern but does not identify which specific mechanisms, trial design optimization, endpoint selection, analytical choices, peer review, or publication bias, drive the observed enrichment.

Concordant-positive prevalence

Among the 77 statistically significant empirical trials, 30 met the CP criteria, yielding a prevalence of 39.0% (95% CI = 28.8-50.1%). Relative to the null-simulation expectation of 1.4%, this represents a 27.9-fold elevation (exact binomial p < 0.0001).

This pattern, statistically significant results that are simultaneously stable and strongly separated from neutrality, is not captured by partial evidence reporting. For example, a trial with p = 0.04, fragile classification (MFQ = 0.02), and weak robustness (RQ = 0.05) is reported identically to one with p = 0.04, stable classification (MFQ = 0.35), and strong robustness (RQ = 0.55). Both are simply “statistically significant.” The p-fr-nb framework evidence suggests that the fragile, weakly robust result may warrant caution regarding reproducibility, whereas the stable, strongly robust result provides more convincing evidence. Empirical validation of these metrics as predictors of replication outcomes remains important future work.

Critically, the observed 39.0% prevalence substantially exceeded simulation expectations even for strong true effects. The simulation maximum of 4.7% (at RR = 0.60) suggests that random trial generation, even with strong effects, rarely produces the combination of stability and strong robustness observed in published trials. The empirical prevalence of 39.0% is 8.3-fold higher than the simulation maximum, indicating that published trials are enriched for high-quality statistical evidence patterns compared to what unfiltered random data generation produces. This enrichment likely reflects trial design optimization, reporting standards, peer review filtering, and potentially publication bias, rather than the intrinsic superiority of published findings.

Component analysis

Among 77 statistically significant trials, the p-fr-nb framework revealed substantial heterogeneity invisible to p-values alone. The majority of significant trials were fragile, with 47 of 77 trials (61.0%) having MFQ ≤ 0.10. Only 30 (39.0%) trials demonstrated stable classifications (MFQ > 0.10). The median MFQ was 0.052 (IQR = 0.025-0.225), indicating that half of all significant trials required outcome changes in only 5.2% or less of one arm to flip significance.

Robustness patterns showed different heterogeneity. Nearly one-third of significant trials (24/77, 31.2%) exhibited weak robustness (RQ < 0.075), indicating that the observed data lay close to neutrality despite achieving statistical significance. Moderate robustness (RQ = 0.075-0.227) occurred in 17 (22.1%) trials, while strong robustness (RQ ≥ 0.227) was observed in 36 (46.8%) trials. The median RQ was 0.175 (IQR = 0.055-0.390).

Among statistically significant trials, the CP pattern was present in 30/77 (39.0%); a fragile with weak or moderate robustness was present in 41/77 (53.2%); and a fragile with strong robustness was present in 6/77 (7.8%). The SFW pattern was present in 24/77 (31.2%) of statistically significant trials. All statistically significant trials that were stable were also strongly robust (30/77, 39%).

Comparison of empirical distributions to simulation expectations revealed substantial enrichment for both stability (39.0% empirical vs. 1.4% in null simulation, representing 27.9-fold elevation) and strong robustness (46.8% empirical vs. 10.9% in null simulation, representing 4.3-fold elevation).

Nonsignificant trials

Among the 52 nonsignificant trials, 10 (19.2%; 95% CI = 10.8-31.9%) showed concordant null evidence, defined as p > 0.05, MFQ > 0.10, and RQ < 0.075. In these studies, a stable, nonsignificant p-value with geometric proximity to neutrality points in the same direction: the data are most compatible with no meaningful effect. Stability (MFQ > 0.10) occurred in 57.7% (30/52) of nonsignificant trials overall, and strong robustness (RQ ≥ 0.227) was present in 5.8% (3/52). Simulation results indicate that this concordant-null pattern appears at similar frequencies under null and non-null scenarios (range = 21.4-28.8%), so this is treated as a descriptive marker of strong null-compatible evidence, given p > 0.05, that is, evidence that clearly points toward no meaningful effect in this dataset, rather than as a tool for deciding whether the true effect is precisely zero.

## Discussion

Among 77 statistically significant clinical trial results in this heterogeneous sample, the p-fr-nb evidence revealed two sharply contrasting categories hidden behind the uniform label “p ≤ 0.05.” In 30/77 (39%) trials, the findings were not only significant but also demonstrated high stability (resistant to small outcome changes) and strong geometric separation from neutrality, the CP pattern representing high-quality statistical evidence. Yet, standard p-value reporting makes these indistinguishable from the remaining 47/77 (61%) trials that did not meet the CP criteria, including 24/77 (31.2%) trials occupying a precarious boundary zone where significance is fragile and observed effects lie close to no effect at all (the SFW pattern). Partial evidence alone leaves readers unable to distinguish convincing from precarious, boundary-zone evidence.

The reproducibility crisis in medical research stems partly from treating all p ≤ 0.05 results as equivalent [17]. These findings demonstrate they are not. This p-fr-nb framework provides p-value stratification through two complementary metrics. Fragility quantifies how easily significance classifications reverse with small outcome changes, essentially asking “how many outcome changes would flip the p-value across the 0.05 threshold?” Fragility ranges continuously from 0 (fragile) to 1 (stable), enabling cross-study comparisons and systematic meta-analyses.

Robustness quantifies geometric distance from therapeutic neutrality, where treatment equals control. It directly addresses “how strong is the evidence that a real, non-zero effect exists?”: a question that effect sizes and confidence intervals answer only indirectly and without standardization across studies. Robustness ranges from 0 (at neutrality) to 1 (far from neutrality), providing a consistent scale for assessing evidence strength. Together with the p-value, fragility and robustness form a statistical evidence triplet that stratifies findings in ways partial evidence cannot.

The simulation revealed a concerning pattern: approximately one-quarter of all statistically significant findings occupy a boundary zone where results are technically significant (p ≤ 0.05) yet fragile (easily flipped by small outcome changes) and close to neutrality (minimal separation from no effect). This SFW pattern occurred at similar rates whether the underlying effect was strong, moderate, or absent entirely, meaning it marks a precarious region of the evidence space rather than identifying “false positives.” Current p-value reporting provides no mechanism to flag these boundary-zone results. A reader seeing p = 0.04 with 95% CIs can assess effect size and precision, but cannot determine whether significance is fragile or stable, nor do they have a standardized measure of distance from neutrality across diverse study designs. Statistical evidence that includes fragility and robustness makes this distinction immediately visible, allowing readers to quickly identify statistically significant results that warrant caution, replication, or closer scrutiny before clinical implementation.

Both fragility and robustness metrics are computed directly from published 2×2 tables using freely available software, with framework extensions to continuous, ordinal, survival, and correlation analyses available though not yet empirically validated beyond binary outcomes [14]. While computational burden is low, implementation requires additional statistical literacy and may face barriers in routine clinical research settings without dedicated statistical support. The incremental reporting burden is minimal: two additional numbers alongside the p-value. The inferential gain is substantial: readers can immediately assess whether a significant finding represents convincing evidence (high stability, strong robustness) or boundary-zone evidence warranting caution (low stability, weak robustness). While the present validation focused on binary outcomes, the framework includes metrics for continuous, ordinal, survival, and correlation analyses; empirical validation of these extensions represents important future work. Incorporating these metrics would provide readers with an assessment of significance, classification stability, and distance from neutrality.

This study has limitations. The trial sample was obtained through pragmatic PubMed searches rather than a systematic review of a single clinical question, which could introduce selection bias. However, sampling was conducted across 15 clinical specialties with diverse populations, and the CP prevalence appeared consistent across domains. This validation is restricted to binary outcomes analyzed as independent-sample 2×2 tables; generalizability to continuous, ordinal, survival, and correlation outcomes requires empirical validation. Additionally, extreme sample sizes may disproportionately influence RQ values, and this study does not directly link p-fr-nb metrics to replication outcomes.

While the observed enrichment for high-quality patterns is consistent with effective filtering throughout the trial pipeline, from design optimization through peer review, the specific steps that drive this enrichment cannot be determined by this study. The data demonstrate the collective outcome but cannot isolate individual contributions.

Fragility and robustness thresholds were derived empirically from simulation distributions; different thresholds would yield different prevalence estimates, though sensitivity analyses indicated that substantially lower thresholds weakened discrimination.

The central finding remains robust despite these limitations: published significant trials show evidence quality patterns that random trial generation rarely produces. The 27.9-fold elevation in CP prevalence demonstrates systematic enrichment for stability and robustness beyond what chance alone predicts. This enrichment likely arises from multiple factors: optimized trial design (power analysis driving sample size selection), strategic endpoint selection, analytical flexibility, peer review filtering, and potentially publication bias. The simulation-to-empirical comparison serves as descriptive benchmarking, documenting what patterns published trials contain versus what unfiltered random data generation produces, not as evidence of the inherent superiority of published trials or bias correction by the framework. The framework stratifies evidence quality within published trials; it does not validate the publication process itself. Yet, current reporting standards obscure this critical heterogeneity entirely, treating a fragile, boundary-zone result as if it were convincing evidence. As the reproducibility crisis continues to challenge the credibility of medical research, the addition of fragility and robustness evidence offers a practical, implementable solution for assessing evidence quality. The framework is validated, and the software is available. These findings support future evaluation by reporting guideline bodies such as CONSORT regarding potential incorporation of fragility and robustness metrics for binary outcome trials.

## Conclusions

In this pragmatic observational study of 129 two-arm, binary-outcome trials, statistically significant findings exhibited broad underlying heterogeneity in both fragility and robustness. Among findings with p ≤ 0.05, about 40% were both stable and strongly robust. In contrast, about 30% of these significant results showed high fragility and weak robustness. More than half of the significant trials showed at least one of these vulnerabilities (high fragility or weak robustness), features that are not apparent from p-values alone. Nonsignificant findings were also separated into distinct patterns, with about 20% of trials confirming the p-value classification by showing stable results close to neutrality, and the rest showing instability or stronger separation from the neutrality boundary. These results demonstrate that fragility and robustness metrics provide interpretive dimensions not captured by p-values alone, enhancing the assessment of evidence heterogeneity among statistically significant and nonsignificant findings.
